# Resveratrol Inhibits Nucleosome Binding and Catalytic Activity of PARP1

**DOI:** 10.3390/biom14111398

**Published:** 2024-11-02

**Authors:** Daria O. Koshkina, Natalya V. Maluchenko, Anna N. Korovina, Angelina A. Lobanova, Alexey V. Feofanov, Vasily M. Studitsky

**Affiliations:** 1 Department of Bioengineering, Faculty of Biology, Lomonosov Moscow State University, 12, Leninskie Gory, Moscow 119234, Russia; koshdar@ro.ru (D.O.K.); angelina260503@mail.ru (A.A.L.); 2 Shemyakin and Ovchinnikov Institute of Bioorganic Chemistry, Russian Academy of Sciences, ul. Miklukho-Maklaya, 16/10, Moscow 117437, Russia; 3 Cancer Epigenetics Team, Fox Chase Cancer Center, Cottman Avenue 333, Philadelphia, PA 19111, USA

**Keywords:** poly(ADP-ribose) polymerase-1, PARP1, nucleosome, resveratrol, spFRET, Western blotting, gel-shift analysis, absorption spectroscopy

## Abstract

The natural polyphenol resveratrol is a biologically active compound that interacts with DNA and affects the activity of some nuclear enzymes. Its effect on the interaction between nucleosomes and poly(ADP-ribose) polymerase-1 (PARP1) and on the catalytic activity of PARP1 was studied using Western blotting, spectrophotometry, electrophoretic mobility shift assay, and single particle Förster resonance energy transfer microscopy. Resveratrol inhibited PARP1 activity at micro- and sub-micromolar concentrations, but the inhibitory effect decreased at higher concentrations due to the aggregation of the polyphenol. The inhibition of PARP1 by resveratrol was accompanied by its binding to the enzyme catalytic center and a subsequent decrease in PARP1 affinity to nucleosomal DNA. Concurrent binding of talazoparib to the substrate binding pocket of PARP1, which occurs in the presence of resveratrol, restores the interaction of PARP1 with nucleosomes, suggesting that the binding sites of resveratrol and talazoparib overlap. The data suggest that resveratrol can be classified as a natural inhibitor of PARP1.

## 1. Introduction

Poly(ADP-ribose) polymerase-1 (PARP1) is a nuclear enzyme that is involved in DNA repair, replication, and transcription, cell cycle regulation, and apoptosis. Dysregulation of PARP1 activity and expression leads to pathological processes [1]. Thus, the increased level of PARP1 expression correlates with the severity of various oncological diseases [2,3,4,5]. Furthermore, higher levels of PARP1 expression are characteristic of tumor stem cells that are resistant to anti-cancer therapies [6,7,8].

In oncology, the negative role of PARP1 is most likely related to its genome guardian function. Increased expression of PARP1 is necessary for more efficient DNA damage repair due to the increased instability of the nuclear structure during uncontrolled tumor cell division and/or after radiation/chemotherapy treatments. In addition, PARP1 regulates a number of important processes that directly affect tumor growth, such as metabolic, angiogenetic, and proliferation pathways [9]. PARP1 might contribute to oncogenesis by NAD+-dependent inhibition of SIRT1, which leads to significant impairment of mitochondrial biogenesis with a shift of cellular processes toward glycolytic metabolism. A shift from traditional oxidative respiration to aerobic glycolysis (Warburg effect) is one of the known hallmarks of the carcinogenic process [10,11,12,13]. Inhibition of SIRT1 by PARP1 also promotes tumor angiogenesis [14].

PARP1 increases the expression of reactive oxygen species and exerts potent pro-inflammatory effects through activation of NF-κB [15]. Lack of PARP1 inactivation can lead to a depletion of macroenergetic molecules (NAD+ and ATP) in cells [16]. In this case, cells may undergo necrosis and PARthanatos, which are processes involved in the development of many diseases, including neurodegeneration and neuroinflammation, as well as various cardiovascular and metabolic disorders [15].

Accordingly, therapies aimed at pharmacological inhibition of PARP1 are considered promising for both tumor treatment and the therapy of neurodegenerative, diabetic, and cardiovascular diseases. Several FDA-approved PARP1 inhibitors, initially proposed for use in tumor treatment, have also demonstrated therapeutic effects in models of non-cancerous diseases such as stroke, Parkinson’s disease, Alzheimer’s disease, multiple sclerosis, diabetes, and myocardial infarction [17,18,19,20,21,22]. The main challenge with the widespread use of PARP1 inhibitors is their high level of toxicity and the many associated side effects [23]. This is why the search for new inhibitors remains a priority.

Some plant polyphenols, including myricetin, quercetin, epigallocatechin, fisetin, tricetin, gossypetin, and delphinidinm, have been shown to inhibit the activity of PARP1 [24,25,26]. In this regard, searching for new PARP1 inhibitors having lower toxicity from a vast group of natural plant polyphenols represents a promising approach. The natural polyphenol resveratrol (3,5,4′-trihydroxystilbene, RSV) has a wide range of activities, including antioxidant, anti-inflammatory, antibacterial, antiviral, geroprotective, and antitumor therapeutic activities [27,28,29,30,31]. It provides cardio-, neuro-, and hepatoprotection and improves cognitive function [32,33,34]. RSV has been found to affect the functioning of many nuclear enzymes, including DNA polymerase α, topoisomerase II, NAD+-dependent deacetylase (SIRT1), DNA methyltransferase, ribonucleotide reductase, adenosine monophosphate kinase, and transcription factor NF-kB [35,36,37,38]. RSV interacts with DNA [39,40,41], but this interaction does not cause a significant disturbance in the nucleosome structure [31].

In this paper, we report on the effect of RSV on the interaction of PARP1 with nucleosomes and its catalytic activity. It is shown that RSV binds to the catalytic center of PARP1 and reduces the binding of the enzyme to nucleosomal DNA at micro- and sub-micro-molar concentrations. This hinders enzyme activation and inhibits the poly-(ADP-ribose)ylation (PARylation) reaction.

## 2. Materials and Methods

### 2.1. Reagents

RSV of 98% purity was supplied by Roth (Karlsruhe, Germany). RSV (100 mM) was dissolved in dimethylsulfoxide (DMSO) and stored at −20 °C. Before the experiments, this solution was fourfold diluted with distilled water. Talazoparib (TAL, Selleck, Houston, TX, USA) was dissolved in DMSO at 10 mM, stored at −20 °C, and diluted before experiments to have 1 μM in water solution containing 25% DMSO. Recombinant human histones H2A, H2B, H3, and H4 and human PARP1 were obtained as described previously [42]. Fluorescently labeled double-stranded DNA (187 bp) containing nucleosome-positioning sequence 603-42A [43] flanked by two fragments of 20 bp length was obtained as described previously [31,44]. Fluorescent labels cyanine 3 (Cy3) and cyanine 5 (Cy5) were placed at positions 13 and 91 bp, counting from the boundary of the nucleosome-positioning sequence. Nucleosomes were assembled as described previously [45].

### 2.2. spFRET Microscopy

Nucleosomes (1–2 nM) were pre-incubated with different concentrations of RSV (1, 10 or 100 μM) for 20 min in low adhesion tubes in TB150 buffer (20 mM Tris-HCl (pH 7.5), 5 mM MgCl_2_, 150 mM KCl, 1 mM β-mercaptoethanol) at room temperature (RT), mixed with PARP1 (40 nM) and incubated for 30 min at RT. In experiments with TAL, PARP1 (40 nM) was pre-incubated with RSV (10 μM) for 20 min, mixed with nucleosomes (2 nM) and TAL (1 μM), and incubated for 30 min in TB150 buffer at RT. Alternatively, PARP1 was initially incubated with TAL, followed by incubation with nucleosomes and RSV (the same concentrations and incubation times were used).

spFRET measurements were performed using the LSM710-Confocor3 laser scanning confocal microscope (Zeiss, Oberkochen, Germany), as described earlier [46]. Fluorescence was excited at 514.5 nm and recorded using avalanche photodiodes in the 530–635 nm (Cy3 fluorescence) and 635–800 nm (Cy5 fluorescence) ranges. The fluorescence intensities of Cy3 and Cy5 measured from single nucleosomes and their complexes with PARP1 were used to calculate proximity ratios (E_PR_) as described previously [43]. E_PR_ is the FRET efficiency without correction for quantum yields of fluorescent labels and sensitivity of the detection system. The sets of E_PR_ values (the sample sizes were >2500 values) were used to calculate the frequency distributions of nucleosomes and their complexes with PARP1 by E_PR_ (E_PR_ profiles). The calculated E_PR_ profiles were averaged over at least three independent experiments and approximated by a linear superposition of several Gaussian peaks.

### 2.3. Electrophoretic Mobility Shift Assay (EMSA)

Complexes of nucleosomes with PARP1 and RSV were prepared for use in EMSA studies in the same manner as for spFRET microscopy. Electrophoresis in the 5% polyacrylamide gel was performed at 100 V in 0.2×TBE buffer (3.6 mM Tris (pH 7.5), 3.6 mM boric acid, and 0.08 mM EDTA) for 1–1.2 h at +4 °C. The gel was pre-run before the sample loading. Fluorescence analysis of the gels was performed using the Amersham Typhoon RGB laser scanner (GE Healthcare, Chicago, IL, USA) at the 532 nm excitation wavelength, detecting the fluorescence of the Cy3 label.

### 2.4. Western Blot Analysis

Nucleosomes (2 nM) were pre-incubated with RSV (1 nM–100 μM) in TB150 buffer at RT for 20 min, incubated with PARP1 (40 nM) for 10 min, supplemented with β-nicotinadenine dinucleotide (NAD+, 20 μM, Sigma, St. Louis, MO, USA) and incubated for 45 min at RT. Then, the samples were heated to 95 °C for 5–10 min after the addition of plating buffer (5× buffer: 312 mM Tris-HCl, pH 6.8, 10% SDS, 25% β-mercaptoethanol, 0.05% bromphenol blue) and applied to the 4–12% gradient gel (Mini-protean gels TGX, Hercules, CA, USA, Bio-Rad). Electrophoresis was performed in Tris-glycine buffer (25 mM Tris-HCl, 192 mM glycine, and 0.1% SDS, pH 8.6) at 130 V for 1 h at RT. Protein transfer to a nitrocellulose membrane (Bio-Rad) was performed in the transfer buffer (50 mM MOPS, 50 mM Tris-HCl, 1 mM EDTA, 3.5 mM SDS) containing 20% ethanol at 4 °C (350 mA, 2 h). Then, the membrane was incubated for 1 h in PBS-T solution (2.7 mM KCl, 8 mM Na_2_HPO_4_, 2 mM KH_2_PO_4_, 37 mM NaCl, 0.5% Tween 20) supplemented with 5% skimmed milk and washed twice with PBS-T solution for 5 min each. The membrane was incubated with mouse monoclonal antibodies against polyADP-ribose (10H, ab14459, Abcam, Waltham, MA, USA) for 1 h in PBS-T solution containing 5% milk, washed, incubated for 1 h with horseradish peroxidase-conjugated secondary anti-mouse antibodies (Bio-Rad, Hercules, CA, USA) and washed again. Imaging of the membrane was performed using the ChemiDoc system (Bio-Rad) and the SuperSignal™ West Pico PLUS chemiluminescent substrate (Thermo Fisher Scientific, Waltham, MA, USA). Data of Western blotting analysis were reproduced in three independent experiments. Original western blots can be found at Supplementary Materials.

### 2.5. UV–VIS Spectrophotometry

Absorption spectra of RSV (0.1–100 µM) were measured in an aqueous solution containing 25% dimethyl sulfoxide using a NanoDrop™ 2000c spectrophotometer (Thermo Fisher Scientific, Waltham, MA, USA) in the 250–400 nm spectral range. The spectra recorded in two independent experiments were averaged.

## 3. Results

**Figure 1:**
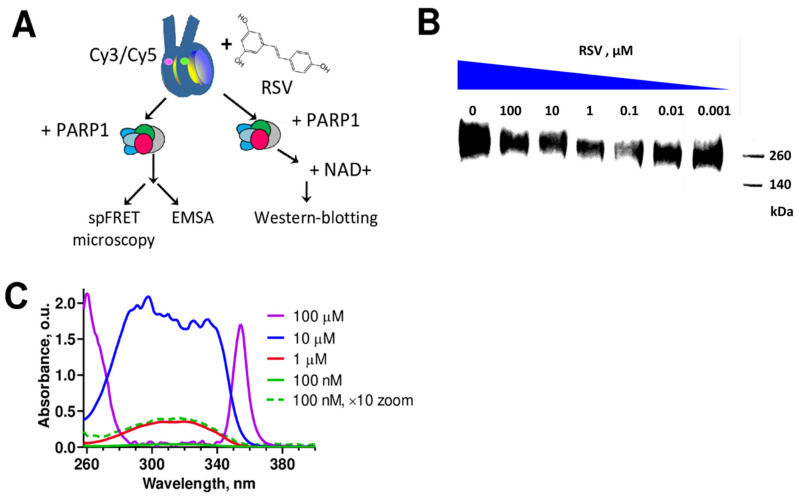
Effect of RSV on the interaction between PARP1 and nucleosomes and on PARP1 activity. (**A**) The experimental approach. Small pink and green circles show the positions of fluorescent labels Cy3 and Cy5 on the nucleosomal DNA. Different domains of PARP1 are shown in color to highlight the multidomain structure of the enzyme. (**B**) Western blotting analysis of the effect of RSV on the PARP1 activity. Antibodies to poly(ADP-ribose) chains were used to stain auto-PARylated PARP1 after nucleosome-activated reaction of PARylation in the mixture of nucleosomes (Nuc, 2 nM), PARP1 (40 nM), NAD+ (20 μM) and different concentrations of RSV. M: protein molecular weight markers. Similar data were obtained in three independent experiments. (**C**) Absorption spectra of RSV at different concentrations in an aqueous solution containing 25% dimethyl sulfoxide. The dashed line shows the RSV spectrum at 100 nM magnified ×10 times in intensity.

The effect of RSV on the binding and catalytic activity of PARP1 in chromatin was studied using nucleosomes (basic structural units of chromatin) as a model system. Nucleosomes and their complexes with PARP1 formed in the presence of RSV were analyzed using the Western blotting technique, spFRET microscopy, and EMSA (Figure 1A).

### 3.1. RSV Inhibits Catalytic Activity of PARP1

PARP1 is activated after its binding to DNA or to histones H3 and H4 [47]. Catalytically active PARP1 utilizes NAD+ as a substrate to synthesize poly-(ADP-ribose) chains of various lengths and structures. These chains are covalently attached to neighboring proteins and the enzyme itself (auto-PARylation) [48,49]. The binding of PARP1 to nucleosomes also leads to its activation and NAD+-dependent auto-PARylation [50]. Accordingly, Western blotting using antibodies to poly(ADP-ribose) chains reveals the accumulation of heterogeneous in molecular weight auto-PARylated PARP1 in the solution containing nucleosomes, PARP1, and NAD+ (Figure 1B). The addition of RSV to this solution prior to the addition of NAD+ leads to concentration-dependent changes in the intensity of the poly(ADP-ribose)-related signal as detected by the Western blotting (Figure 1B). The signal related to poly(ADP-ribose) is minimal at the 0.1 μM concentration of RSV, and it increases both at lower and, surprisingly, at higher concentrations of RSV.

The data suggest that RSV inhibits PARP1 at micro- and sub-micro-molar concentrations, but its inhibitory effect becomes weaker at concentrations above 1 μM. A possible explanation for the decreased PARP1 inhibition at higher concentrations of RSV is the aggregation of RSV molecules in an aqueous solution.

### 3.2. RSV Aggregates in an Aqueous Solution

To evaluate the possibility of RSV aggregation in a water-based solution, UV–VIS spectra were measured at different concentrations of RSV (Figure 1C). At concentrations of RSV below 1 μM, the intensity of light absorption is directly proportional to the RSV concentration, and the shape of the absorption spectrum remains unchanged. The increase in RSV concentration above 1 μM is not accompanied by a proportional increase in light absorption, and the shape of the spectrum changes dramatically (Figure 1C). Both observations indicate that the aggregation of RSV occurs in the 10–100 μM concentration range. Microscopic examination of an aqueous solution of RSV revealed the formation of precipitating needle-like crystals when the RSV concentration was greater than 100 μM.

The spectrophotometric data (Figure 1C) suggest that the decrease in the ability of RSV to inhibit PARP1 at concentrations above 1 μM (Figure 1B) is caused by the aggregation of RSV in solution.

### 3.3. RSV Reduces the Binding of PARP1 to Nucleosomes

To further study the PARP1 inhibition by RSV, the formation of complexes between PARP1 and nucleosomes was examined in the presence of RSV using spFRET microscopy and EMSA.

**Figure 2:**
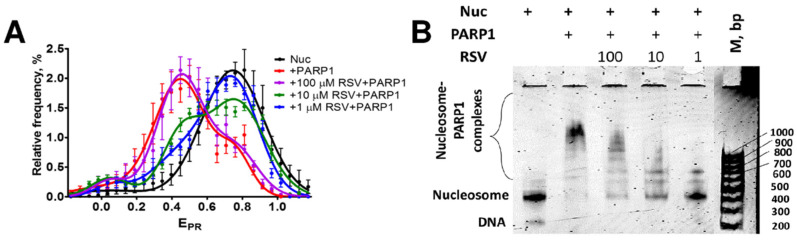
RSV reduces the binding of PARP1 to nucleosomes. (**A**) E_PR_ profiles of nucleosomes (Nuc) and their complexes with PARP1 (40 nM) in the absence and presence of RSV (1, 10 and 100 μM). E_PR_ profiles are presented as mean ± standard error of the mean (n = 3). (**B**) EMSA data for nucleosomes and nucleosome–PARP1 complexes in the absence and presence of RSV (1, 10, and 100 μM).

In agreement with the published results [51], the binding of PARP1 to nucleosomes causes structural changes in nucleosomal DNA, which are detected by spFRET microscopy as a shift of the peak of the nucleosome distribution in the E_PR_ profile from high (~0.75) to lower (~0.49) E_PR_ values (Figure 2A). RSV itself minimally affects the structure of nucleosomes and does not cause any changes in the E_PR_ profile of nucleosomes labeled at positions 13 and 91 bp at the concentrations of RSV ranging from 1 to 100 μM [31]. The addition of RSV to nucleosomes alters the interaction between PARP1 and nucleosomes (Figure 2A). RSV reverses the changes in the E_PR_ profile of nucleosomes induced by PARP1 at RSV concentrations of 1 or 10 μM. This effect is not observed at an RSV concentration of 100 μM (Figure 2A) and can be attributed to the extensive aggregation of RSV (Figure 1).

EMSA confirms that RSV disturbs the interaction between PARP1 and nucleosomes (Figure 2B). As was shown earlier, PARP1 can form several types of complexes with nucleosomes that differ in the number of PARP1 molecules bound to each nucleosome [51]. The formation of PARP1–nucleosome complexes is significantly reduced at an RSV concentration of 1 μM (Figure 2B). However, the formation of complexes between PARP1 and nucleosomes is progressively restored with an increase in the RSV concentration from 10 to 100 μM (Figure 2B). This observation is likely explained by the concentration-dependent aggregation of RSV (Figure 1).

Taken together, spFRET microscopy and EMSA data suggest that the inhibition of PARP1 by RSV at micro- and sub-micro-molar concentrations is likely related to the RSV-mediated decrease in the ability of PARP1 to bind to nucleosomes and become activated.

### 3.4. RSV Binds to the Catalytic Center of PARP1

The inhibiting effect of RSV could be explained by either the binding of RSV at the interface of interaction between PARP1 and a nucleosome or by the binding of RSV within the catalytic center of PARP1, similar to some other inhibitors of PARP, which also decrease the affinity of PARP1 to DNA and nucleosomes [31,52]. Inhibitors of PARP1 that bind to the catalytic center of the enzyme are classified into three groups based on their ability to modify the affinity of PARP1 to DNA. These groups include inhibitors that increase affinity (type I), decrease affinity (type III), and have no effect on the affinity of PARP1 for DNA (type II) [53]. This classification of the inhibitors also applies to the interaction between PARP1 and nucleosomal DNA [54].

**Figure 3:**
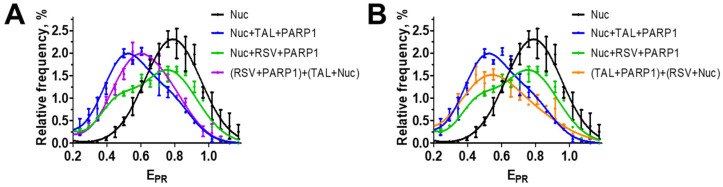
Effect of RSV and TAL on the interaction between PARP1 and nucleosomes. E_PR_ profiles of nucleosomes (Nuc) and their complexes with PARP1 (40 nM) in the absence and presence of RSV (10 μM) and TAL (1 μM). E_PR_ profiles are presented as mean ± standard error of the mean (n = 3). (**A**) PARP1 was pre-incubated with RSV and then incubated with nucleosomes and TAL. (**B**) PARP1 was pre-incubated with TAL and then incubated with nucleosomes and RSV.

To evaluate the hypothesis about RSV binding to the catalytic center of PARP1, we studied the interaction between PARP1, nucleosomes, and RSV in the presence of talazoparib (TAL) using spFRET microscopy. TAL is a PARP1 inhibitor that binds to the catalytic center of PARP1 and increases the affinity of the enzyme to DNA [52,53] and nucleosomes [55]. The addition of 1 µM TAL together with nucleosomes to the mixture of PARP1 and RSV restores the interaction between PARP1 and nucleosomes, overcoming the inhibiting effect of RSV. This is evident by the shift of the peak of the nucleosome distribution in the E_PR_ profile from higher E_PR_ values characteristic of intact nucleosomes to lower E_PR_ values characteristic of PARP1–nucleosome complexes (Figure 3A). A similar effect was observed when TAL was first mixed with PARP1 and then added to a mixture of nucleosomes and RSV (Figure 3B). This result suggests that RSV reversibly binds to the catalytic center of PARP1. TAL competes with RSV for the binding to the catalytic center, displacing RSV from the center and increasing the efficiency of the formation of the complex between PARP1 and nucleosomes.

## 4. Discussion

The data suggest that RSV is an inhibitor of PARP1. The highest inhibitory activity is achieved at 100 nM RSV, but it decreases at higher concentrations due to aggregation of RSV in an aqueous solution (Figure 1B). Inhibition of PARP1 is caused by the binding of RSV in the catalytic center of the enzyme, as was demonstrated in the experiments addressing competitive binding of TAL (Figure 3A,B). The data suggest a mechanism involving blocking the binding of NAD+ substrate in the active center of PARP1 and the inhibition of its catalytic activity. The RSV binding weakens the interaction between PARP1 and nucleosomal DNA, suppressing the activation of PARP1 (Figure 2A,B). This effect is typical for type III PARP1 inhibitors, which, by binding to the catalytic center, stabilize the helical domain of the enzyme and change the interactions between the domains of the enzyme, reducing its affinity to DNA [52].

Alternatively, it could be proposed that RSV binds somewhere at the interface of the interaction of PARP1 with DNA, interfering with the formation of the complex. According to in vitro experiments, RSV forms a complex with DNA, but the dissociation constant of the complex is in the 52–180 µM range [39,40]. Thus, RSV does not bind to DNA in the 0.1–1 µM concentration range, in which inhibition of PARP1 is observed. The restoration of the interaction between PARP1 and nucleosomes in the presence of TAL supports this conclusion and allows us to rule out RSV binding to another site on PARP1 other than the catalytic center, causing disruption of the complexes between PARP1 and nucleosomes.

To our knowledge, inhibition of PARP1 by RSV is reported for the first time. RSV expands the list of natural polyphenols, such as myricetin, quercetin, fisetin, tricetin, gossipetin, and delphinidin, which were reported to inhibit PARP1 [24,56]. The activity of RSV exceeds, for example, the activity of quercetin, which inhibits PARP1 at micromolar concentrations [57].

RSV can probably be useful as an inhibitor of unwanted PARP1 hyper-activation in the prevention and/or in the treatment of some metabolic and inflammatory diseases such as cardiovascular, Parkinson’s, and Alzheimer’s diseases, neurodegenerative disorders, and diabetes [15,20,58].

The high PARP1-inhibitory activity of RSV makes it a promising candidate for the therapy of breast and ovarian cancers having the susceptibility gene mutation (BRCAm), where the use of synthetic PARP1 inhibitors is currently recommended.

RSV, like some other natural polyphenols, has multiple beneficial effects on the body. Therefore, even though RSV was not previously known as a proven inhibitor of PARP1, trials have been initiated to explore the potential of combining RSV with clinically approved PARP1 inhibitors. After the combined application of RSV and olaparib, deregulation of the homologous recombination (HR) pathway and an increase in apoptosis were observed in breast cancer cells that have a BRCA wild phenotype with undisturbed HR repair. This combination of compounds does not have restrictions on the use of olaparib as a stand-alone drug, as this inhibitor has previously only been used to treat cancer patients with BRCA1 mutation and HR deficiency. RSV was shown to effectively sensitize talazoparib-induced cell death in BRCA wild-type breast cancer cells (possessing undisturbed HR). It was found that RSV caused disruption of cell cycle regulation and enhanced talazoparib-induced double-strand breaks (DSBs), leading to abnormal mitotic progression, culminating in mitotic catastrophe [59]. The reported effects could be at least partially related to the PARP1 inhibitory activity of RSV; a detailed study is necessary to assess the contribution of RSV to the inhibition of PARP1 in vivo when combined with other PARP1 inhibitors. It should be noted that the action of type III inhibitors is gentler than that of type I and II inhibitors, as they do not cause strong toxicity or severe side effects in patients. Apparently, the combined use of various PARP1 inhibitors, one of which is a natural polyphenol with an overall beneficial effect on the body, could be a promising approach to the treatment of tumors.

The concentration-dependent aggregation of RSV reported by us probably can contribute to the dichotomous effect of RSV that was observed previously [60,61,62]. In the clinical trial, high doses of RSV (2000 mg/day) in patients with Alzheimer’s disease were found to lead to increased levels of beta-amyloids and worsening brain volume loss [63]. However, another clinical trial in Alzheimer’s disease showed the beneficial effects of RSV using low doses (5 mg/day) [64]. RSV has been shown to have neuroprotective effects at low concentrations but can be neurotoxic at higher concentrations [61]. Similarly, the opposite effects have been observed with high and low doses of RSV in cardiovascular studies [65,66,67].

## 5. Conclusions

Many natural polyphenols are biologically active compounds that have a variety of health benefits. This has led to numerous attempts to explore their potential not only as nutritional supplements, but also as potential drugs or components for adjuvant therapy. In line with this, the findings reported in the present study lead to the conclusion that further clinical trials of combinations of RSV and clinically approved anticancer drugs targeting PARP1 should take into account the direct inhibiting effect of RSV on PARP1. Additionally, RSV itself should be investigated as a potential anticancer agent targeting PARP1.

## Data Availability

The data presented in this study are available on request from the corresponding authors. The data are not publicly available due to local regulations.

## Supplementary Materials

The following supporting information can be downloaded at: https://www.mdpi.com/article/10.3390/biom14111398/s1, Original western blots.
