# Supplementary material for: Resveratrol Inhibits Nucleosome Binding and Catalytic Activity of PARP1
Source: Biomolecules. 2024 Nov 2;14(11):1398. doi: 10.3390/biom14111398 (PMC11591765; doi:10.3390/biom14111398)

Fig. 1b western blotting

1. original blot without editing

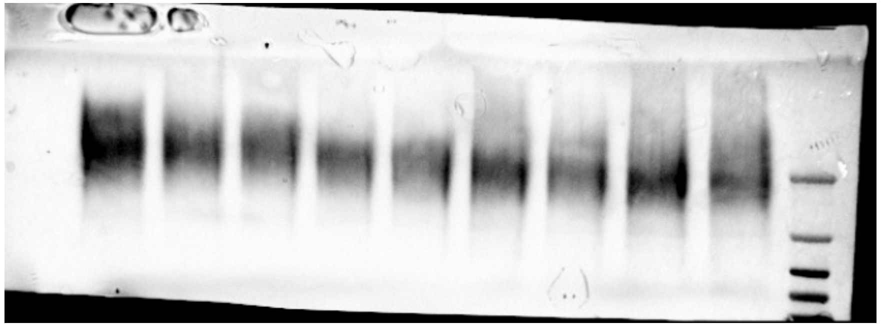

2. original blot with light contrast

3. blot for fig 1b

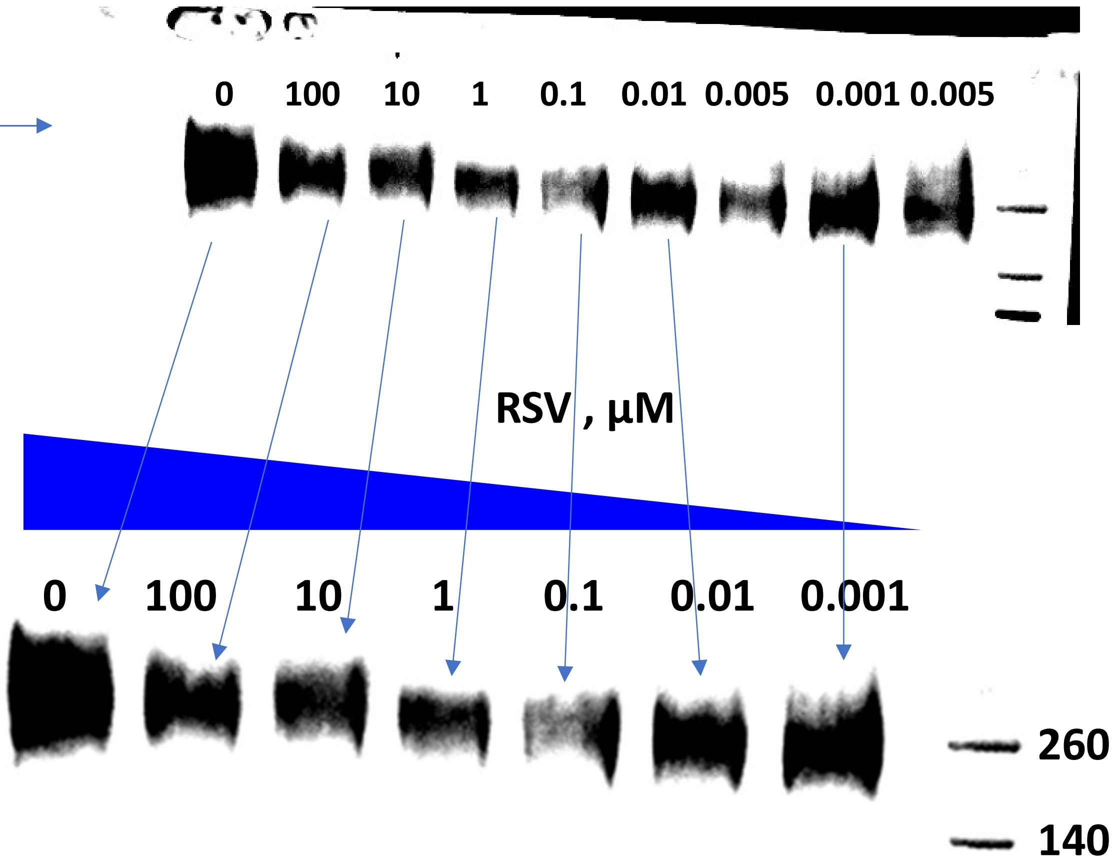

Fig. 2b gel-shift

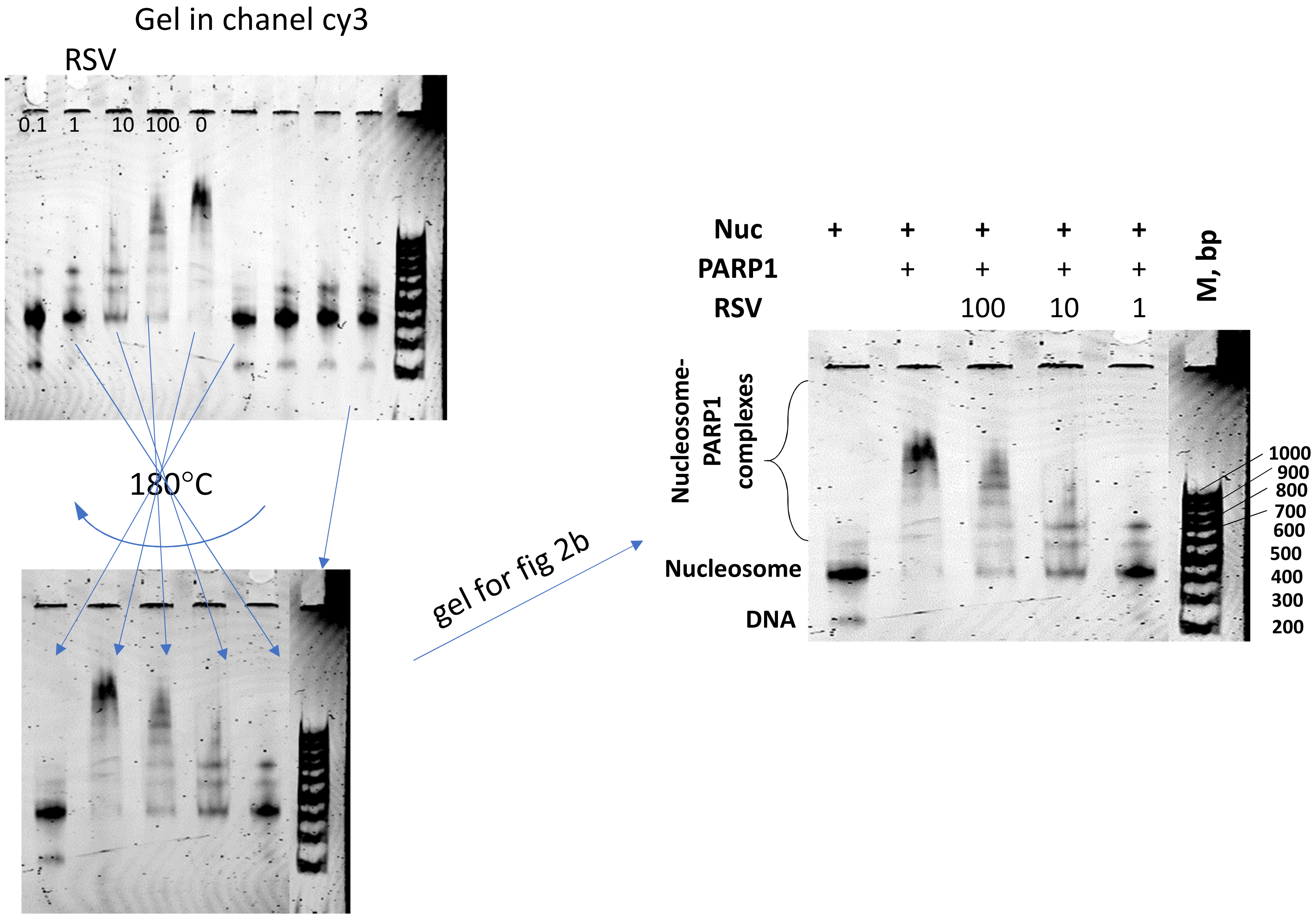

Supplement: Supplementary file 1 [file biomolecules-14-01398-s001.zip › biomolecules-3257195.pdf]
